# Use of biomarkers to differentiate 4‐repeat tauopathies from AD

**DOI:** 10.1002/alz70856_101936

**Published:** 2025-12-25

**Authors:** Indira Ruth Garcia Cordero, Gabor G. Kovacs, Blas Couto, Alonso Morales‐Rivero, Carmela Tartaglia

**Affiliations:** ^1^ Tanz Centre for Research in Neurodegenerative Diseases, University of Toronto, Toronto, ON, Canada; ^2^ Universidad de San Andres, Victoria, Buenos Aires, Argentina; ^3^ Department of Laboratory Medicine and Pathobiology, University of Toronto, Toronto, ON, Canada; ^4^ Rossy PSP Program, University Health Network and the University of Toronto, Toronto, ON, Canada; ^5^ Laboratory of Progressive Neuromotor and Neurobehavioral Disorders at Instituto de Neurociencia Cognitiva y Traslacional (INCYT), CABA, Buenos Aires, Argentina; ^6^ Unidad de Movimientos Anormales, Institute of Neuroscience of the Fundacion Favaloro, CABA, Buenos Aires, Argentina; ^7^ Toronto Western Hospital, University Health Network Memory Clinic, Toronto, ON, Canada; ^8^ Toronto Western Hospital, Tanz Centre for Research in Neurodegenerative Disease, Toronto, ON, Canada; ^9^ Canadian Concussion Centre, Toronto Western Hospital, University Health Network, Toronto, ON, Canada

## Abstract

This session examines how Alzheimer's disease (AD)‐related biomarkers influence the clinical presentation, brain structure, and functional connectivity in patients with corticobasal syndrome (CBS) and progressive supranuclear palsy (PSP). Although a few descriptions have associated certain clinical features to CBS and PSP with AD‐neuropathological changes, those are not completely specific. AD fluid biomarker positivity is associated with distinct patterns of brain atrophy and functional disconnection detected using neuroimaging technics (MRI and fMRI), as well as differences in motor and cognitive features. Those results highlight the importance of studying the presence of AD co‐pathology to understand how it may shape the clinical and neuroimaging manifestations of CBS and PSP. This session will provide insights into how biomarker‐based stratification of patients can enhance diagnostic accuracy and inform personalized approaches to the management of these complex syndromes.

